# Levofloxacin for the treatment of severe refractory BK virus-associated hemorrhagic cystitis in hematopoietic stem cell transplantation recipients: A report of three cases

**DOI:** 10.3892/ol.2014.2381

**Published:** 2014-07-25

**Authors:** TAYFUR TOPTAS, ISIK KAYGUSUZ-ATAGUNDUZ, HALUK TARIK KANI, CAFER ADIGUZEL, TULIN FIRATLI-TUGLULAR

**Affiliations:** 1 Department of Hematology, Van Training and Research Hospital, Van 65100, Turkey; 2 Department of Hematology, Marmara University Hospital, Istanbul 34890, Turkey

**Keywords:** BK virus, hemorrhagic cystitis, levofloxacin, hematopoietic stem cell transplantation

## Abstract

BK-virus (BKV) is an important etiological agent for late-onset hemorrhagic cystitis (HC) in patients undergoing hematopoietic stem cell transplantation. Late-onset HC causes significant morbidity among these patients. Therapeutic approaches remain predominantly symptomatic. Several treatment options have been used with variable success rates. Cidofovir has the highest specificity against BKV; however, its lack of availability in the majority of countries, high costs and potential nephrotoxic effects limit its use. The present study reports three cases of severe and prolonged BKV-associated HC (BKHC). HC was resolved in all three of the patients using oral levofloxacin. Thus, levofloxacin may be an effective treatment modality for achieving complete clinical and molecular response in patients with refractory, severe BKHC.

## Introduction

Viral infections are important causes of morbidity and mortality following allogeneic hematopoietic stem cell transplantation (HSCT). Patients who have undergone transplantation are susceptible to primary viral infections and to the reactivation of latent viruses, including the polyomavirus BK virus (BKV) (1).

Hemorrhagic cystitis (HC) is characterized by hemorrhagic inflammation of the bladder mucosa, leading to painful micturition with hematuria. HC is commonly associated with immunosuppression caused by chemotherapy, radiotherapy or HSCT. Clinically, HC ranges from mild and brief (Grade I) to severe, prolonged and life threatening (Grade IV). Although BKV has been found to be an important etiological agent for late-onset HC, until now, therapy has been predominantly symptomatic with disappointing results achieved using conventional antiviral drugs (2).

The present study reports the clinical and molecular activity of levofloxacin in patients with severe BK-associated hemorrhagic cystitis (BKHC) that is refractory to ciprofloxacin and other supportive measures. Patients provided written informed consent.

## Case report

### Patient presentation

The present study describes the cases of three patients who presented with severe BKHC within 100 days of undergoing HSCT. The patients received a full myeloablative conditioning regimen and routine ciprofloxacin prophylaxis (500 mg, orally) twice daily from the day of stem cell infusion until neutrophil engraftment (Table I). In September 2006, patient 1, a 40-year-old male, was diagnosed with standard cytogenetic risk acute myelogenous leukemia (AML) at the Marmara University Hospital (Istanbul, Turkey). In 2008, following 3+7 (12 mg/m^2^/day idarubicin on days one to three, and 100 mg/m^2^/day cytarabine on days one to seve, for one cycle) induction and high dose cytarabine (3 g/m^2^ every 12 h on days one, three and five) consolidation chemotherapy (three cycles), the patient underwent autologous HSCT as the patient did not have a matched donor. By post-transplant day 77, the patient experienced gross hematuria. In February 2008, patient 2, a 37-year-old male was treated with 3+7 and high dose cytarabine chemotherapy due to standard risk AML at the Marmara University Hospital. In October 2008, the patient underwent a sibling donor HSCT. Gross hematuria was observed on post-transplant day 49. Patient 3, a 45-year-old female, was referred to Marmara University Hospital following a sibling donor HSCT following a diagnosis of standard risk AML at the Transplantation Unit, Erciyes University Hospital (Kayseri, Turkey) in 2008. The patient reported gross hematuria on post-transplant day 48. All patients presented with gross hematuria, as well as dysuria and painful micturition.

### Diagnosis

Routine microscopic analyses and bacterial cultures revealed no specific pathogenic organisms. Furthermore, analyses for adenovirus, cytomegalovirus, mycoplasma and ureaplasma were all negative. However, BKV was detected to varying degrees using reverse transcription polymerase chain reaction (RT-PCR) analysis in the urine of the patients. Adenovirus, cytomegalovirus, mycoplasma, BKV and ureaplasma-associated HC were included in the differential diagnosis.

### Treatment

All of the patients exhibited severe HC which required continuous intravesical irrigation due to gross hematuria and urinary obstruction caused by blood clots. All patients received ciprofloxacin for two weeks, as well as intravesical instillation of risperidone in order to control the bleeding. Hyperbaric oxygen and subsequent external iliac artery embolization were performed in one patient; however, no improvement was observed. All patients were treated with levofloxacin (500 mg a day, orally), which was administered for eight weeks, and urine BKV copies were monitored every four weeks.

### Follow-up

**Figure 1:**
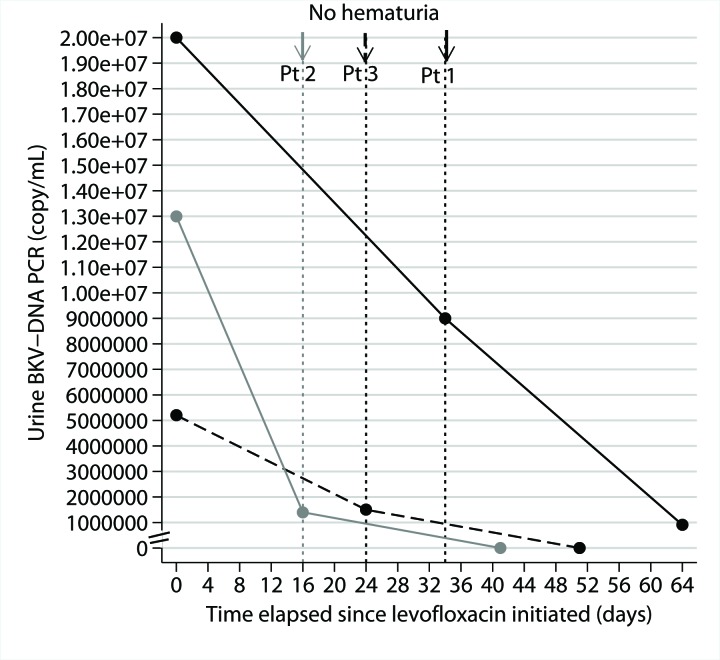
BKV copies in the urine of patients with BK-associated hemorrhagic cystitis. BKV, BK virus; Pt, patient; PCR, polymerase chain reaction.

In patient 1, HC resolved completely after one month of levofloxacin treatment. The other two patients also received the same treatment and complete clinical responses were achieved. The BKV copies in the urine were found to decrease by >90% at the end of the eight weeks of levofloxacin treatment (Fig. 1).

## Discussion

BKV is a member of the Polyomaviridae family of viruses. BKV was first isolated in 1971 in the urine of a patient who was asymptomatic and immunocompromised (3). Primary infection with BKV usually occurs during childhood and is generally asymptomatic. Thereafter, the virus remains latent in the host. BKV affects the epithelia of the renal pelvis, ureter and urinary bladder. Common routes of transmission have been proposed to be through respiratory or fecal spread (2).

BKHC is defined by the co-occurrence of HC with BK-viruria, which is detected using urinary RT-PCR analysis targeting the BKV VP1 gene (4). The presence of blood clots as a result of macroscopic hematuria, with or without urinary retention, is defined as severe HC. Severe BKHC is reported in ~15% of patients with BKHC and causes significant morbidity among these patients (5).

Cidofovir has the highest specificity against BKV. The majority of the evidence comes from non-randomized or retrospective studies in renal transplantation settings. However, its lack of availability in the majority of countries, high costs and potential nephrotoxic effects limit the use of cidofovir. Estrogens, leflunomide, hyperbaric oxygen, clotting factors (for example factor VII and XIII), intravesical instillation of saline, prostaglandin, risperidone, aluminum, formalin, growth factors, immunosuppressive dose reduction, selective arterial embolization and cystectomy have been used to treat patients with BKV, with variable success rates. However, the majority of these procedures have high costs, significant morbidity and limited activity (2,6).

The *in vitro* and *in vivo* activity of ciprofloxacin on BKV in prophylactic use has been shown by a previous study with certain limitations (7). However, data with regard to the efficacy of levofloxacin in patients with BKHC are limited in prophylactic and therapeutic settings. Levofloxacin and other third generation quinolones have high *in vivo* activity against intracellular atypical pathogens compared with ciprofloxacin; thus, levofloxacin may also have a unique *in vivo* antiviral activity.

The current study presents three post-HSCT patients fulfilling severe HC criteria, which was refractory to several interventions mentioned in the literature previously (2,6). Cidofovir is an active and safe treatment in post-HSCT BKV-associated HC (8). However, Cidofovir was not administered as it is not registered in Turkey. The activity of levofloxacin in the setting of severe active refractory HC can be explained by the probable antiviral effect of levofloxacin. Two recent studies exploring the activity of levofloxacin on BK-viremia in renal transplant recipients exhibited conflicting results (9,10). A retrospective study, including 40 patients who received levofloxacin or ciprofloxacin, reported that a one month course of fluoroquinolones, used with the intention to treat other bacterial infections within three months following renal transplant, was associated with a significant risk reduction of one-year BK-viremia (9). However, a more recent prospective, placebo-controlled, double-blinded, randomized trial including 39 patients with post-renal transplant BK viremia failed to verify such an effect (10). To the best of our knowledge, this is the first report to demonstrate the activity of levofloxacin in the setting of refractory BK-associated HC in post-HSCT patients. Since the plasma BK viral load was not measure, a conclusion can not be determined with regard to its effect on viremia. However, we hypothesize that levofloxacin exerts its main activity on the urothelial epithelium by inhibiting BK-viral replication, which results in symptomatic improvement and reduction of BK-viruria. However, this hypothesis remains to be elucidated in prospective studies.

Considering the high morbidity of severe BKHC and the potential drawbacks of currently available treatment options, levofloxacin (500 mg a day, orally) may present as an effective treatment modality for achieving complete clinical and molecular response in patients with refractory, severe BKHC. The use of levofloxacin may prevent costly and invasive procedures.

## Figures and Tables

**Table I:** Characteristics of patients with with BKHC.

Patient	Diagnosis: FAB/Cytogenetic risk group	Gender	Age (years)	HSCT	Post-HSCT day	IS/GVHD	Therapies given for HC	IS dose reduction
1	AML-M2/intermediate	M	40	Autologous	+77	NA/none	Ciprofloxacin, IV risperidone	NA
2	AML-M7/intermediate	M	37	Matched sibling, MA	+49	cSA/none	Ciprofloxacin, IV risperidone	None
3	AML-M4/intermediate	F	45	Matched sibling, MA	+48	cSA/none	Ciprofloxacin, IV risperidone, HOT, EIAE	None

HSCT, hematopoietic stem cell transplantation; IS, immunosuppressive drug; GVHD, graft versus host disease; HC, hemorrhagic cystitis; MA, myeloablative; cSA, cyclosporine; HOT, hyperbaric oxygen therapy; EIAE, external iliac artery embolization; NA, not applicable; BKHC, with BK-associated HC; IV, intravenous; AML, acute myeloid leukemia; FAB, French-American-British.
